# Effects of Different Types of Starch on Physicochemical Properties and Microstructure of Beef during Cold Storage

**DOI:** 10.3390/foods13172767

**Published:** 2024-08-30

**Authors:** Shulin Zhang, Lina Wang, Qiuyu Wang, Yuqi Wang, Linlin Wang, Rongsheng Du

**Affiliations:** 1 College of Food Science and Technology, Southwest Minzu University, Chengdu 610041, China; 17371823406@163.com (S.Z.); wlmdsciggyx@163.com (L.W.); akkko_858@163.com (Q.W.); wyq010728@hotmail.com (Y.W.); 2 School of Food and Biological Engineering, Hefei University of Technology, Hefei 230601, China; 3 Lu’an Soyea Electrical Manufactring Co., Ltd., Lu’an 237000, China; 4 Sichuan Institute of Musk Deer Breeding, Chengdu 610016, China

**Keywords:** beef gel, microstructure, physicochemical properties, starch

## Abstract

The purpose of this study was to identify the most effective method for enhancing the quality of beef gel during refrigeration. To achieve this objective, the effects of various types of starch on the physicochemical properties and microstructure of beef gel during refrigeration were investigated. In this study, ground beef gel was chosen as the research subject, and six different types of starch were added: 6% tapioca starch, cassava-modified starch (acetylated distarch phosphate, ADSP), potato starch (PSP), modified potato starch (acetate starch, SA), corn starch (CSP), and modified corn starch (hydroxypropyl distarch phosphate, HPDSP). The quality indicators of ground beef were measured and analyzed throughout the cold storage at 4 °C on days 1, 3, 5, 7, and 9. The results demonstrated that the water capacity of beef mince supplemented with PSP and HPDSP was significantly greater (*p* < 0.05). Additionally, the gel strength was found to be the highest, while the mesh structure formed in the ADSP group was the greatest. Furthermore, HPDSP, PSP, and SA effectively inhibited the oxidation of meat fat, with SA showing a relatively good effect on delaying the oxidation of meat mince protein. The addition of starch can, to a certain extent, inhibit lipid and protein oxidation in meat mince. In conclusion, starch significantly enhances the quality of beef mince by improving water retention, gel strength, and microstructure during refrigeration.

## 1. Introduction

Beef is characterized by a high protein content of approximately 22%, with a fat content of around 10%. The ratio of n-6/n-3 polyunsaturated fatty acids (PUFAs) is favorable, and it also contains various minerals including phosphorus, potassium, and sodium, as well as vitamins such as vitamin E, thiamin, and riboflavin [1]. Beef is a commonly consumed type of red meat, and its demand is significant worldwide [2]. As the primary processed product of beef, ground beef is used to create more refined food products for consumer consumption. Currently, the main ground meat products include sausages, meatballs, and ham, among others. Ground beef retains the original nutrients found in beef while offering several advantages. It has a softer texture, with finer and easily separable meat fibers, which facilitates easier digestion and absorption by the human body. This makes ground beef particularly suitable for the elderly and children with weaker digestive functions. However, a significant amount of meat juice is often lost during the processing of meat, leading to considerable reductions in water retention, nutrition content, sensory quality, and other factors. This loss severely impacts product market development and the economic benefits for enterprises [3]. Wang et al. [4] found that the addition of starch can reduce the cost of meat products, significantly improve their gel properties, and enhance their freeze–thaw stability. Eshag Osman et al. [5] found that starch possesses highly favorable functional attributes, including the enhancement of meat viscosity, moisture retention capacity, water-holding capacity, gelation ability, and emulsion stability. Additionally, the quality of meat products is likely to deteriorate due to oxidation occurring during processing and storage. Protein oxidation affects various attributes, including color, tenderness, gel formation, and flavor [6]. Oxidation can lead to the disruption of the secondary and tertiary structures of proteins, as well as the breaking and folding of amino acid chains. These processes manifest as protein carbonylation, desulfhydrylation, and cross-linking [7].

Tapioca starch, extracted from cassava roots, is recognized for its cost-effectiveness, high yield, and straightforward extraction methods [8]. Over the years, tapioca starch has been extensively utilized in various food applications as a gelling agent, thickener, water-retaining agent, stabilizer, and filler. This is attributed to its lower point, higher viscosity, and superior shear resistance in comparison to starch from other sources [9] Nelum Pematilleke et al. [10] have demonstrated that the addition of tapioca starch to beef patties can enhance their texture and facilitate swallowing. Acetylated distarch phosphate (ADSP) is a modified starch created through the cross-linking of natural starch with sodium trimetaphosphate or phosphorus oxychloride, followed by esterification with acetic anhydride. ADSP provides high acid resistance and thermal stability, which helps prevent the degradation of starch molecules during storage, making it a popular choice in meat processing [11]. In a study conducted by Li et al. [12], the incorporation of ADSP as a pre-emulsifier in emulsified minced fish sausage resulted in enhanced water retention, improved emulsification stability, and increased whiteness performance.

Potato starch (PSP) is a type of starch extracted from potatoes through a series of processing steps. Fenghui Zhang et al. [13] have found that the incorporation of potato starch can reduce the cooking loss in beef gel formulations. This process effectively helps prevent the quality deterioration of surimi gel during freeze–thaw cycles. Acetate starch (SA), also known as acetylated starch, is a modified starch produced by esterifying the hydroxyl group of starch macromolecules with acetic acid or other acid derivatives under specific conditions. Bo Cui et al. [14] have demonstrated that the acetylation of potato starch can enhance its rheological properties and strengthen the gel structure.

Corn starch (CSP) is a natural polysaccharide derived from the endosperm of corn grains [15]. In a study by Wu et al. [16], it was observed that corn starch can influence the relaxation peak area of the starch–pork myofibrillar protein complex gel, leading to a reduction in relaxation time and an improvement in water holding capacity. Hydroxypropyl distarch phosphate (HPDSP) is a modified starch produced through a combination of phosphoric acid crosslinking and hydroxypropyl ether modification. This double-modified starch combines the benefits of hydroxypropyl starch and crosslinked starch. The addition of hydroxypropyl groups aids in maintaining hydration and transparency, while crosslinking contributes to enhanced thermal, acid, and shear stability [17]. Studies have suggested that HPDSP can form a complex with egg yolk-modified starch, functioning as a novel emulsifier, or interact with shrimp myofibrillar protein to enhance the quality of shrimp mince gel products [18,19].

Moreover, many studies have concentrated on the effect of a single type of starch on the storage of meat products, while relatively few have compared the effects of different types of starch on beef gels.

Therefore, the aim of this study was to identify the optimal method for improving the quality of beef gel during refrigeration. Specifically, 6% cassava flour, ADSP, PSP, SA, CSP, and HPDSP, respectively. A total of 6% of cassava flour, ADSP, PSP, SA, CSP, and HPDSP were added. The gelatin quality indices of beef minced meat were determined and analyzed at 4 °C for 1, 3, 5, 7, and 9 days. The effects of different types of starch on the physicochemical properties and microstructure of meat mince were investigated. This study provides data support and a theoretical basis for the future research and development of meat products and the enhancement of product quality.

## 2. Materials and Methods

### 2.1. Materials and Equipment

Beef hind leg meat was bought in Baijia market, Shuangliu District, (Chengdu, China); NaCl (Huazhong Biotechnology Co., Ltd., Jinan, China); Na_2_HPO_4_, NaH_2_PO_4_, ethylene glycol tetraacetic acid (EGTA), MgCl_2_, sodium dodecyl sulfate (SDS), urea, 5,5′-dithio bis-(2-Nitrobenzoic acid, DTNB), 2,4-dinitrophenylhydrazine (2,4-Dinitrophenylhydrazine, DNPH), trichloroacetic acid, ethanol, ethyl acetate, guanidine hydrochloride, bromophenol blue, copper sulfate, potassium sodium tartrate, sodium hydroxide were all analytical pure (Chengdu Kelon Chemical Reagent Factory, Chengdu, China). Tapioca, acetylated distarch phosphate (ADSP), potato starch (PSP), acetate starch (SA), corn starch (CSP), hydroxypropyl distarch phosphate (HPDSP) (Xintai Biotechnology, Shanghai, China)Portable pH meter was from Ingenieurhuro R. Matthaus (Berlin, Germany), and CR-400 chromatic aberrator was purchased from Konica minolta (Tokyo, Japan), TA.XT. Plus Texture analyzer was purchased from Stable Micro System (London, UK); Waters LLC Rheometer was purchased from New Castle, (DE, USA); F-4700 fluorophotometer was purchased from Hitachi High Tech Science Nake Corporation (Tokyo, Japan).

### 2.2. Preparation of Minced Beef

The purchased beef underwent initial preparation by removing visible fat and connective tissue from its surface. Subsequently, the beef was cut into small pieces and minced using a meat grinder with a mince dish outlet diameter of 0.5 cm through two grinding cycles. The minced meat was then mixed, portioned into bags (approximately 185 g each), and stored in a refrigerator at 4 °C.

### 2.3. Preparation of Beef Gel

A random small bag of minced meat was treated with 1.0% NaCl and refrigerated at 4 °C for 12 h. Subsequently, 6% starch and 6% deionized water were added to the minced meat, thoroughly mixed, placed into a bag, and steamed at 85 °C for 20 min. The steamed product was then cooled in an ice–water mixture and stored in the refrigerator at 4 °C. Samples were collected on days 1, 3, 5, 7, and 9 during the refrigeration period and allowed to reach room temperature for further testing and analysis.

### 2.4. pH

The pH of the sample was measured using a portable pH meter to assess its acidity level. Insert directly into the meat for measurement. Each sample was measured three times, and the average value was recorded for analysis.

### 2.5. Chroma

The L*, a*, and b* of the surface of the ground meat gel were measured directly with an automatic color difference meter.

### 2.6. Water-Retaining Property

#### 2.6.1. Water Content

Weigh the mass of the bottle containing sea sand and glass rods, as well as the sample (denoted as *m*_1_). Heat the sample for 1 h, then allow it to cool and weigh the mass (denoted as *m*_2_). Subsequently, dry the sample at 101 °C to 105 °C for 2 to 4 h, cool it again, and weigh it. This process should be repeated until the mass difference is less than 2 mg. Finally, weigh the mass of the bottle, including the sea sand and glass rods (denoted as *m*_3_).

(1) $$\mathrm{Water}\;\mathrm{content}\left(\%\right)=\frac{m_{1}\;-\;m_{2}}{m_{1}\;-\;m_{3}}\times 100\%$$

#### 2.6.2. Cooking Loss (CL)

The weight of the polyethylene bag used for cutting the treated meat gel at room temperature (*A*) was recorded. Any water present on the gel surface (*B*) was carefully removed using double paper napkins.

(2) CL(%)=[(A − B)/A]×100%

#### 2.6.3. Non-Expressible Water, NW, and Water-Holding Capacity, WHC

The ground meat gel was cut into slices of 10 mm thickness, designated as its mass *m*_1_. These slices were then sandwiched between two layers of filter paper and subsequently measured for mass after extrusion, referred to as *m*_2,_ using the Hold Until Time test mode of the texture analyzer.

(3) $$\mathrm{NW}\left(\%\right)=100\%\times [1\;-\;(m_{1}-m_{2})/m_{1}]$$

(4) $$\mathrm{WHC}\left(\%\right)=[\left(100\;-\;\mathrm{CL}\right)\times \mathrm{NW}]/100\%$$

#### 2.6.4. Thawing Loss

The cooked minced meat was measured and recorded as *A*, then frozen at −18 °C for 24 h. Subsequently, it was thawed at 4 °C for 12 h. The free water present on the surface was gently wiped off, and the weight after wiping was recorded as *B*. Thawing loss was then calculated using the specified formula.

(5) thawing loss(%)=[(A − B)/A]×100%

#### 2.6.5. Emulsion Stability

The minced sample (*m*_0_) was transferred into a centrifugal tube and subjected to 0, 1, 4, 7, and 10 freezing–thawing cycles. Following this, the sample was heated at 80 °C for 30 min and then centrifuged at 3000× *g* for 5 min to collect the supernatant (*m*_1_). The water content in the supernatant was determined by heating at 105 °C, and the resulting weight was recorded as *m*_2_.

(6) $$\mathrm{Water}\;\mathrm{release}\left(\%\right)=\left(1\;-\;\frac{m_{2}}{m_{1}}\right)\times 100\%$$

(7) $$\mathrm{Fat}\;\mathrm{release}\left(\%\right)=\left(1\;-\;\frac{m_{1}-m_{2}}{m_{1}}\right)\times 100\%$$

(8) $$\mathrm{Total}\;\mathrm{liquid}\;\mathrm{release}\left(\%\right)=\frac{m_{1}}{m_{0}}\times 100\%$$

### 2.7. Texture Profile Analysis (TPA)

The meat samples were cut into specified sizes and evaluated using a texture analyzer. The parameters included a probe diameter of 0.50 cm. From the test force response curve, five indices could be calculated.

### 2.8. Dynamic Rheological Measurement

An appropriate amount of minced meat samples was placed on the test platform, coated evenly, and bubbles were removed. A 40 mm fixture was used for the test with the following parameters: slit gap of 0.5 mm, frequency of 1 Hz, and strain of 0.0025. The sample was heated from 20 °C to 85 °C at a rate of 2 °C/min and maintained at 85 °C for 1 min. The changes in the G’ and G” of the minced meat were recorded as the temperature increased.

### 2.9. Sensory Evaluation

Five experienced evaluators assessed the quality of beef minced gel products across several aspects using a 1 to 10 scale, as referenced in Table 1. A higher score indicated better quality.

### 2.10. Scanning Electron Microscope (SEM)

The composite gel sample was cut into specified pieces, fixed, sealed, and stored in an appropriate environment for 48 h prior to testing.

### 2.11. Fat Oxidation Index

#### 2.11.1. Peroxide Value (POV)

Weigh 3.0 g of minced meat (*m*) and add the mixed solution. Transfer the mixture and add potassium iodide solution, then shake and incubate in the dark. Subsequently, add water and starch solution, and titrate with Na_2_S_2_O_3_ standard solution. Record the volumes *V* and *V*_0_.

(9) $$\mathrm{POV}\left(\frac{\mathrm{mmol}}{\mathrm{kg}}\right)=\frac{\left(V-V_{0}\right)\times c}{2\times m}\times 1000$$

#### 2.11.2. Thiobarbituric Acid Reactive Substances (TBARSs)

A total of 5.0 g of ground beef was combined with 25.0 mL of trichloroacetic acid solution and homogenized in an ice bath at 3000 rpm for 1 min. The resulting mixture was centrifuged at 5000 rpm and 4 °C for 5 min, followed by filtration. Subsequently, 5.0 mL of the filtrate was mixed with thiobarbituric acid, and the mixture underwent heating, cooling, and further analysis. The supernatant was collected, and absorbance at 532 nm (*A*_532_) and 600 nm (*A*_600_) was measured.

(10) $$\mathrm{TBARS}(\mathrm{mg}/\mathrm{kg})=4.65\times (A_{532}-A_{600})$$

### 2.12. Protein Oxidation Index

#### 2.12.1. Surface Hydrophobic

Bromophenol blue solution was integrated into the ground beef sample and thoroughly mixed prior to centrifugation. The resulting supernatant was meticulously collected, diluted to the appropriate concentration, and the absorbance was measured at 595 nm, yielding a value designated as *A*_1_. A similar experimental procedure was conducted for the control group, with its absorbance recorded as *A*_0_ for comparative analysis.

(11) $$\mathrm{Bromophenol}\;\mathrm{blue}\;\mathrm{binding}\;\mathrm{amount}(\mu g)=200\times \frac{A_{0}-A_{1}}{A_{0}}$$

#### 2.12.2. Carbonyl Content

Add 1 mL of a 2 mol/L HCl solution containing 0.02 mol/L 2,4-dinitrophenylhydrazine (DNPH) to 1 mL of a protein solution (2 mg/mL). For the blank control group, add 1 mL of a 2 mol/L HCl solution without DNPH, while keeping the other operations the same. After mixing, incubate the samples at 25 °C for 40 min. Then, add 2.0 mL of trichloroacetic acid (20% mass fraction), mix, and centrifuge at 4 °C for 5 min at 11,000× *g*. Discard the supernatant and precipitate the protein with 1.0 mL of ethanol: ethyl acetate solution (1:1 volume ratio), washing three times. Suspend the protein in 3.0 mL of a 6.0 mol/L guanidine hydrochloride solution and keep it in a water bath at 37 °C for 30 min. The blank group serves as the control. Absorbance is then measured at a wavelength of 370 nm.

#### 2.12.3. Protein Solubility

The minced meat sample was diluted to a concentration of 2 mg/mL(*x*_0_) using a 0.02 mol/L phosphate buffer. Following centrifugation at 5000× *g* for 15 min at 4 °C, the protein mass concentration in the resulting supernatant was measured using the biuret method (*x*_1_).

(12) $$\mathrm{Protein}\;\mathrm{solubility}(\%)=\frac{x_{1}}{x_{0}}\times 100\%$$

#### 2.12.4. Sulfhydryl Content

The protein solution was diluted to a concentration of 2 mg/mL using 0.02 mol/L sodium phosphate buffer at pH 6.25. Subsequently, 1 mL of the diluted solution was extracted, to which 4 mL of a urea SDS solution with a specified composition and 1 mL of DTNB reagent were sequentially added. Notably, the control group was devoid of DTNB. The absorbance at 412 nm was then monitored at a temperature of 25 °C for a duration of 15 min (*A*_412_).

(13) $$\mathrm{Total}\;\mathrm{sulfhydryl}\;\mathrm{content}=\frac{10^{6}A_{412}\times n}{\varepsilon \rho }$$

#### 2.12.5. Dityrosine

The determination of dityrosine content in minced meat was conducted using a fluorescence spectrophotometer. Initially, the minced meat sample was diluted to a concentration of 0.5 mg/mL in 0.02 mol/L phosphate buffer. Following dilution, the sample was subjected to centrifugation at 5000× *g* for 3 min at 4 °C to isolate the supernatant. The fluorescence intensity of the supernatant was subsequently measured at an excitation wavelength of 325 nm and an emission wavelength of 420 nm.

### 2.13. Fourier Transform Infrared (FT-IR) Spectroscopy

The minced meat was freeze-dried, ground, and analyzed using Fourier-transform infrared spectroscopy to determine the protein’s secondary structure. Spectra were recorded over the range of 500–4000 cm^−1^, with a resolution set at 4 cm^−1^ and an accumulation of 32 scans.

### 2.14. Tertiary Structure

The minced meat was diluted to a concentration of 0.1 mg/mL using a 0.02 mol/L phosphate buffer before undergoing fluorescence spectroscopy analysis. The experimental setup involved an excitation wavelength of 295 nm, with the emission spectrum recorded over the range of 300 to 400 nm. Both the excitation and emission slit widths were set at 5 nm.

### 2.15. Statistical Analysis

Statistical analysis of the data was conducted using Excel 2019, while SPSS 27 was employed to perform significance testing, (*p* < 0.05, indicating a significant difference). Each group consisted of seven samples, and each test was repeated three times. Additionally, Origin 2022 was used for data visualization and plotting.

## 3. Results

### 3.1. pH and Chroma

#### 3.1.1. PH

**Figure 1:**
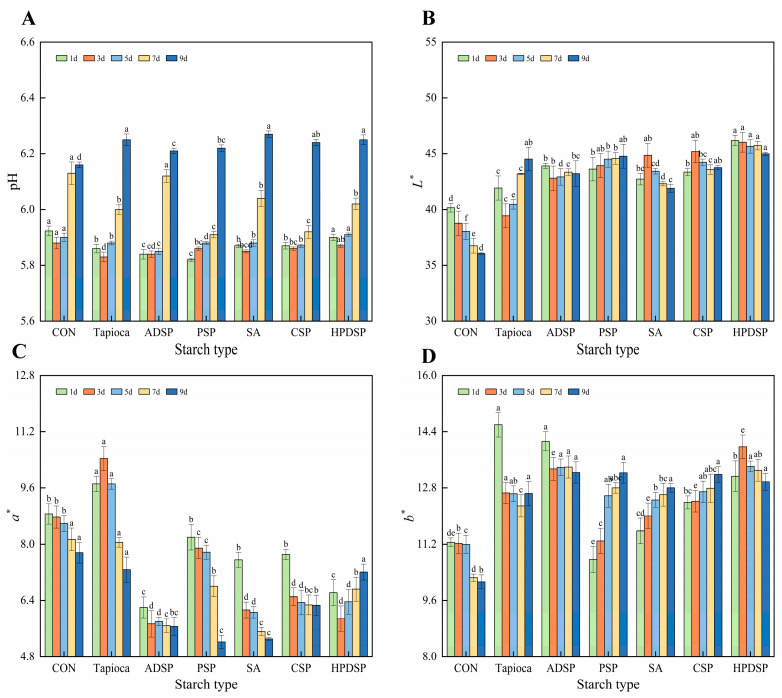
The pH (**A**) and chroma (**B**–**D**) of beef mince were changed under different kinds of starch when it was stored at 4 °C for 1, 3, 5, 7, and 9 days, respectively. Values are the average of values in triplicate ± SD. Different letters indicated significant differences between samples treated with different starch on the same day (*p* < 0.05).

Variations in pH levels were observed over the 1- to 9-day period, as illustrated in Figure 1A. On the 3rd day, the pH levels of minced meat supplemented with tapioca, SA, CSP, and PDSP exhibited a gradual decline (*p* > 0.05). However, as the duration of refrigeration increased, a significant rise in pH was noted (*p* < 0.05). Notably, the minced meat with starch additives presented lower pH levels on the 1st day than the samples without starch. By the 9th day, all starch-supplemented meat groups demonstrated significantly higher pH levels than the non-starch group.

In Figure 1B, observations on the L* of minced meat indicated that the control group had notably lower values than the starch-supplemented samples (*p* < 0.05). Furthermore, L* declined significantly with prolonged refrigeration time (*p* < 0.05). Notably, the L* of mince with tapioca decreased significantly during the first three days, followed by a significant increase thereafter. Similarly, minced meat enriched with SA and CSP exhibited a significant increase in L* on the third day of refrigeration. By the 9th day, the highest L* was recorded for tapioca (44.52) and HPDSP (44.99), significantly surpassing the control group’s value of 36.06.

#### 3.1.2. Chroma

The a* of minced meat treated with tapioca exhibited a significant increase on the 3rd day, followed by a significant decrease from the 3rd to the 9th day (*p* < 0.05). By the 9th day, the a* of starch-supplemented meat was found to be lower than those of the control group. Notably, the a* of meat supplemented with PSP and SA was considerably lower than the control group.

In terms of b*, the control group displayed significantly lower values compared to starch-supplemented minced meat, although a moderate decrease in b* was observed over prolonged refrigeration (*p* > 0.05). The b* of meat enriched with PSP, SA, and CSP exhibited an increase during refrigeration, with higher values recorded for starch-supplemented samples compared to the control group by the 9th day. Notably, ADSP and PSP demonstrated the highest b*, significantly surpassing that of the control group.

Overall, the addition of starch significantly affected the coloration of minced meat during refrigeration. Additionally, PSP and HPDSP notably enhanced and maintained L*. ADSP and HPDSP exhibited the most significant reduction in a*, while all starch types contributed to a notable increase in b*. This indicates a substantial enhancement in the color of minced meat during cold storage.

### 3.2. Water-Retaining Property

#### 3.2.1. Water Content

**Figure 2:**
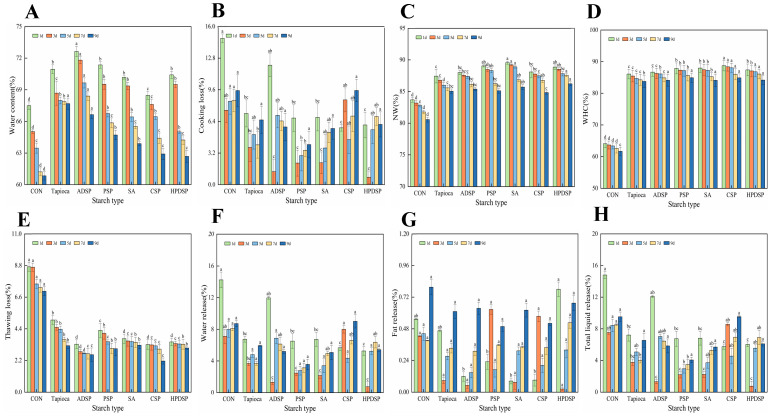
The changes in moisture content (**A**), CL (**B**), NW (**C**), WHC (**D**), freeze–thaw loss (**E**), and emulsification stability (**F**–**H**) of beef mince were observed under different types of starch treatment when it was stored at 4 °C for 1, 3, 5, 7 and 9 days, respectively. Different letters indicated significant differences between samples treated with different starch on the same day (*p* < 0.05).

During refrigeration, the water content of minced meat products supplemented with various types of starch (ADSP, PSP, SA, CSP, HPDSP) and the control group decreased significantly over time, as illustrated in Figure 2A (*p* < 0.05). The minced meat samples enriched with raw or modified starch consistently exhibited higher water content levels compared to the control group throughout the storage period (*p* < 0.05). Notably, on the 9th day, the groups containing tapioca and ADSP recorded water contents of 67.69% and 66.64%, respectively.

#### 3.2.2. Cooking Loss (CL)

Throughout the cold storage period, all minced meat groups initially experienced a significant reduction in water content (*p* < 0.05), followed by a gradual increase. The cooking loss (CL) of starch-supplemented minced meat was notably lower than that of the control group (*p* < 0.01), with the PSP, SA, and HPDSP groups exhibiting significantly lower CL compared to other groups during refrigeration (*p* < 0.05). Particularly, the PSP group displayed the lowest CL on the 9th day, recording a value of 4.06%.

#### 3.2.3. Non-Expressible Water, NW

The NW of minced meat exhibited a general decline during refrigeration, as shown in Figure 2C. Notably, the NW of all starch-supplemented samples was significantly higher than that of the control group (*p* < 0.01), with the modified starch-enriched minced meat showing higher NW compared to the raw starch group. Particularly, on the first day, minced meat samples containing PSP, SA, and HPDSP demonstrated superior NW performance, with NW values of 89.56%, 89.05%, and 88.87%, respectively, significantly surpassing the control group’s value of 83.69%. Even by the 9th day, the NW of starch-enriched minced meat remained higher than that of the control group, at 80.57%.

#### 3.2.4. Water-Holding Capacity, WHC

In Figure 2D, it is evident that all starch-supplemented samples exhibited significantly higher WHC compared to the control samples (*p* < 0.05). The trend of WHC variation in each group mirrored that of CL, displaying a gradual decrease over the refrigeration period. The WHC in starch-supplemented minced meat was notably higher than in the control group (*p* < 0.05), with the PSP and CSP enriched meat mince groups achieving the highest WHC on the 9th day, at 84.91% and 84.89%, respectively.

#### 3.2.5. Thawing Loss

As shown in Figure 2E, along with the prolongation of refrigeration time, the thawing loss of meat in the control group decreased significantly (*p* < 0.01). Notably, the meat samples enriched with added starch consistently exhibited substantially lower thawing losses compared to the control group. Particularly on the first day, ADSP and CSP demonstrated remarkable improvements in freezing–thawing stability, with minimal thawing losses of only 3.31% and 3.33%, respectively. The thawing loss of starch-added meat gel was significantly lower than that of the control group. By the 9th day of refrigeration, the ADSP and CSP mince groups showcased the lowest thawing losses, significantly (*p* < 0.05) outperforming other groups, with values of 2.59% and 2.16%, respectively.

#### 3.2.6. Emulsion Stability

In comparison with the control group, the addition of starch significantly decreased the water release in minced meat (*p* < 0.01). Furthermore, the addition of CSP, ADSP, PSP, and SA significantly reduced the fat release in minced meat (*p* < 0.01). Notably, the water release in the PSP minced meat group reached its lowest level on the 9th day (3.56%). Additionally, the total liquid release from the PSP minced meat group was the lowest on the 9th day (4.05%).

To summarize, starch significantly influences the water retention of minced meat during refrigeration, with the addition of PSP and HPDSP demonstrating the most pronounced effect on enhancing water retention.

### 3.3. Texture Profile Analysis (TPA)

Table 2 illustrates the significant impact of different starch types on the texture of minced meat gel. With extended refrigeration time, the hardness of the mince demonstrated a notable increasing trend. Importantly, the hardness of starch-enriched mince was significantly lower than that of the control group. Specifically, on both the 1st and 9th days, the meat supplemented with ADSP exhibited the lowest hardness, measuring 872 g and 1568 g, respectively. These values were considerably lower than those of the control group (2493 g and 2954 g). This indicates that ADSP had the most pronounced effect on reducing meat hardness.

The addition of SA significantly enhances the springiness of the mince, while other starches tend to reduce the springiness to varying degrees. As refrigeration time is extended, the overall trend indicates a decline in the springiness of the mince. On the 1st day, the springiness of the minced meat was ranked in the following order: SA > control group > tapioca > PSP > CSP > HPDSP > ADSP. Notably, the springiness of the mince supplemented with ADSP was significantly lower than that of the other minced meat samples, with a measured springiness value of 0.65.

The addition of starch effectively decreases the cohesiveness of minced meat. All minced meat samples with added starch exhibited lower cohesiveness than the control group. On the 1st day, the cohesiveness of the minced meat was ranked as follows: control group > SA > CSP > tapioca = PSP > HPDSP > ADSP. Notably, the cohesiveness of the minced meat gel with ADSP was measured at 0.46. As refrigeration time was prolonged, the cohesiveness of the minced meat demonstrated an increasing trend. On the 9th day, the cohesiveness was ranked as follows: control group > CSP > PSP > ADSP = SA = HPDSP > tapioca, with the lowest cohesiveness observed in the group containing tapioca starch, which measured only 0.52. The adhesiveness of all minced meats supplemented with starch was lower than that of the control group, indicating that the addition of starch significantly reduces the adhesiveness of the meat. On the first day, the adhesiveness of the minced meat was ranked as follows: control > tapioca > SA > PSP > CSP > HPDSP > ADSP. As refrigeration time was extended, the adhesiveness of the ground meat exhibited an increasing trend. By the 9th day, the adhesiveness ranking shifted to control > PSP > CSP > SA > tapioca > HPDSP > ADSP. Notably, ADSP significantly decreased the adhesiveness of the minced meat, with values recorded at 401 g·s and 883 g·s on the 1st and 9th days, respectively, which were markedly lower than the control group’s values of 1716 g·s and 2357 g·s.

The addition of starch to minced meats significantly reduced chewiness compared to the control group, highlighting the potential of starch supplementation in decreasing meat chewiness. By the 9th day, the order of chewiness in minced meats was as follows: control group > PSP > CSP > SA > tapioca flour > HPDSP > ADSP. The chewiness values for meat gels enriched with ADSP on the 1st and 9th days were recorded at 260 g and 696 g, respectively, which were markedly lower than those of the control group, which measured 1424 g and 1816 g. These findings suggest that ADSP has a pronounced effect on reducing meat chewiness.

In summary, the texture of minced meat gel during storage is significantly influenced by the addition of various types of starch. Among the various starches, ADSP demonstrates the most substantial influence on the hardness, springiness, adhesiveness, and chewiness of minced meat, while tapioca has the greatest effect on the cohesiveness of minced meat.

### 3.4. Dynamic Rheological Measurement

**Figure 3:**
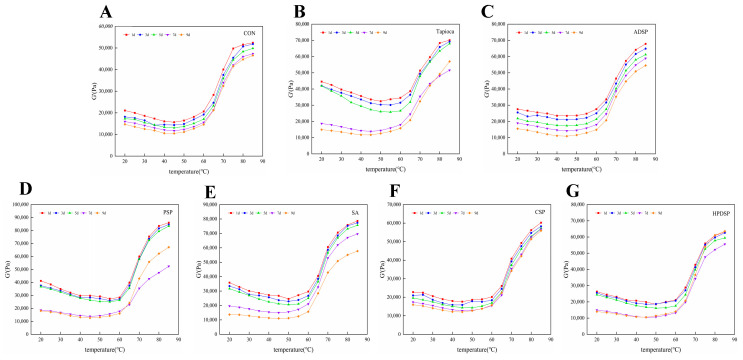
(**A**–**G**) Effects on energy storage patterns of beef minced meat without starch, tapioca, ADSP, PSP, SA, CSP and HPDSP at 4 °C for 1, 3, 5, 7 and 9 days, respectively (G’).

Figure 3 illustrates that minced meat containing various types of starch exhibited a higher storage modulus (G’) during heating compared to the control group, with tapioca and PSP yielding the best results on the initial day of refrigeration. Over time, the G’ of minced meat gradually decreases with prolonged refrigeration. As the temperature increases, the trend in G’ change for starch-supplemented meat mirrors that of the control group. Specifically, as the temperature rises, the G’ decreases gradually between 20 and 40 °C, slowly increases between 40 and 60 °C, stabilizing during this range, and then increases raidly between 60 and 85 °C.

**Figure 4:**
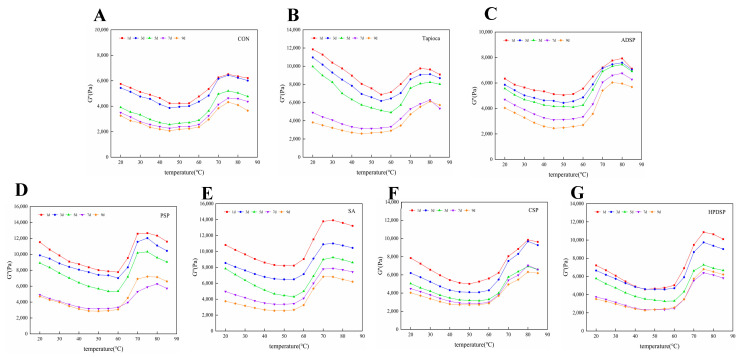
(**A**–**G**) The loss modulus (G’’) of beef mince treated at 4 °C without starch, Tapioca, ADSP, PSP, SA, CSP and HPDSP were changed for 1, 3, 5, 7 and 9 days, respectively.

As shown in Figure 4, the loss modulus (G”) of ground meat with added starch is higher than that of the control group. With prolonged refrigeration time, the adhesiveness of the starch-enriched ground meat gel exhibited a decreasing trend. Compared to Figure 3, the G’ of ground meat is consistently higher than the G”. As the temperature increases, the trend in the strengthening of the G” for the starch-supplemented meat mirrors that of the control group. Specifically, the G” gradually decreases between 20 to 45 °C, stabilizes between 45 to 60 °C, and then increases rapidly between 60 to 75 °C.

### 3.5. Sensory Evaluation and Scanning Electron Microscope (SEM)

#### 3.5.1. Sensory Evaluation

**Figure 5:**
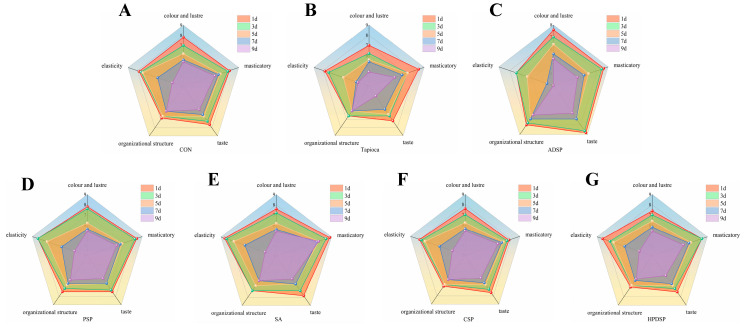
(**A**–**G**) Effects of starch-free, Tapioca, ADSP, PSP, SA, CSP and HPDSP on sensory scores of beef mince stored at 4℃ for 1, 3, 5, 7 and 9 days, respectively.

As shown in Figure 5, the overall sensory evaluation score of beef mince with the addition of tapioca and SA was the highest, while the minced meat gel without any added starch received the lowest score. The gels containing HPDSP excelled in terms of color and taste, while those with tapioca and SA displayed the greatest springiness. Although the quality of beef ground gel with ADSP was superior to that without any starch, its taste, texture, and springiness were slightly inferior to those of CSP. Notably, PSP starch has the least impact on the sensory characteristics of the minced meat gel products.

**Figure 6:**
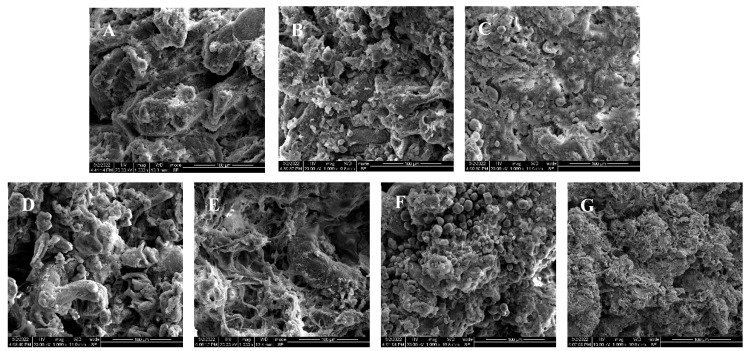
(**A**–**G**) Effects of no starch, Tapioca, ADSP, PSP, SA, CSP and HPDSP on the microstructure of minced meat.

As illustrated in Figure 6, the gel of the control group exhibited a significantly larger pore structure. Following starch treatment, the surface pores of minced meat were significantly reduced (*p* < 0.05). In particular, the minced meat treated with ADSP displayed significantly smaller and more evenly distributed surface pores compared to the other treatment groups (*p* < 0.05). Additionally, the surface shape of the ADSP-treated group was more regular and smooth, resulting in the most optimal network structure among all the groups.

In conclusion, the addition of starch to minced meat significantly influenced its properties when compared to the control group. Notably, the sensory evaluation revealed that the beef minced meat incorporated with tapioca and SA received the highest ratings. The addition of starch not only altered the microstructure of the minced meat gel but also led to the formation of a more robust network structure in the ADSP minced meat group, which demonstrated enhanced water-binding capacity and improved gel water retention.

#### 3.5.2. SEM

In summary, among the ground meat groups, those supplemented with PSP-exhibited HPDSP had the highest G’ during refrigeration, indicating that these two starches positively influenced the gel strength of the ground meat. Additionally, the minced meat group with PSP added showed a higher G” compared to the other groups during refrigeration, indicating that PSP was the most effective in enhancing the adhesiveness of the minced meat.

### 3.6. Lipid Oxidation Index

#### 3.6.1. POV

**Figure 7:**
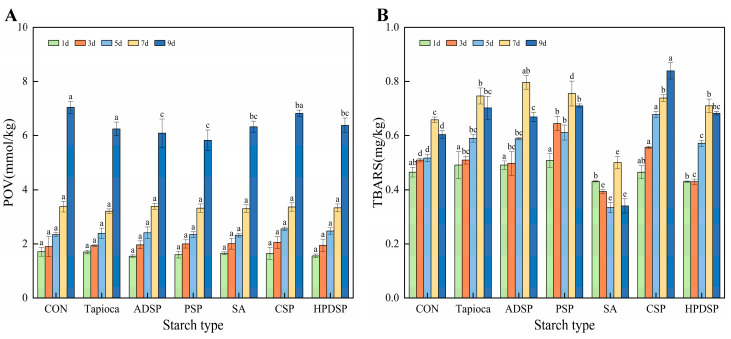
Changes in POV (**A**) and TABRS (**B**) of beef minced meat under different types of starch treated at 4 °C for 1, 3, 5, 7, and 9 days, respectively. Different letters indicated significant differences between samples treated with different starch on the same day (*p* < 0.05).

Figure 7A depicts a progressive increase in the POV of the sample over the storage period, indicating that lipid oxidation occurred in beef mince during storage, which ultimately led to quality deterioration. By the 9th day, the mince supplemented with ADSP and PSP exhibited the lowest POV values of 5.82 mmol/kg and 6.08 mmol/kg, respectively, which were significantly lower than those of the control group (*p* < 0.05). These findings underscore the effective inhibition of fat oxidation conferred by both ADSP and PSP. Furthermore, the addition of SA resulted in a significantly lower POV compared to the control group (*p* < 0.05), indicating its potential to mitigate lipid oxidation in mince to some degree.

#### 3.6.2. Thiobarbiturate Number (TBARS)

In Figure 7B, the TBARS of the sample showed an increasing trend over the storage period, indicating a rise in meat rancidity with prolonged storage. On the first day, the minced meat supplemented with HPDSP and SA exhibited a significantly lower TBARS compared to the other groups (*p* < 0.01), while no significant differences were observed among the remaining groups. Throughout the storage period, the TBARS of mince supplemented with SA remained significantly lower than those of the control group (*p* < 0.05), which is consistent with the POV results. These findings suggest that SA may effectively mitigate lipid oxidation in beef to a certain extent.

In conclusion, the addition of starch demonstrates a measurable influence on delaying the oxidation of meat fat. Among the starch types tested, HPDSP, PSP, and SA exhibited a particularly favorable effect in inhibiting lipid oxidation in meat.

### 3.7. Protein Oxidation Index

#### 3.7.1. Surface Hydrophobic

**Figure 8:**
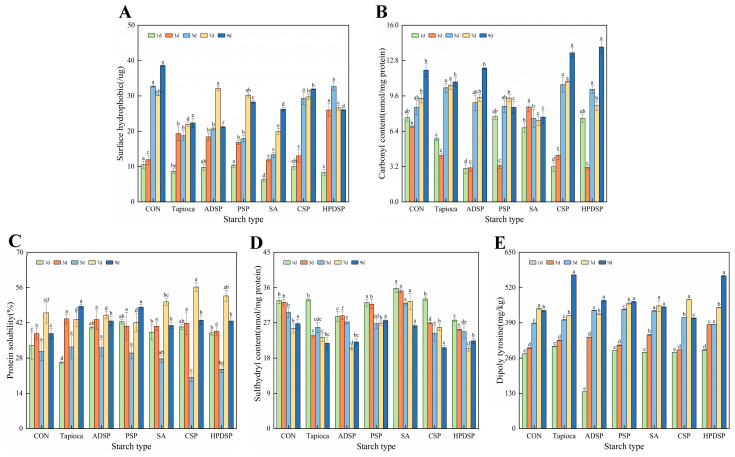
The changes in surface hydrophobicity (**A**), carbonyl content (**B**), protein solubility (**C**), sulfhydryl content (**D**), and dityrosine (**E**) of beef mince were observed under different types of starch treatment when the meat was stored at 4 °C for 1, 3, 5, 7 and 9 days, respectively. Different letters indicated significant differences between samples treated with different starch on the same day (*p* < 0.05).

In Figure 8A, the surface hydrophobicity of minced meat exhibited a general increasing trend during the refrigeration process. Notably, the starch-added groups exhibited lower surface hydrophobicity compared to the control group throughout the refrigeration period, indicating that the addition of starch may mitigate protein oxidation. Specifically, the minced meat supplemented with SA showed significantly lower oxidation levels than the control group for the duration of the storage period (*p* < 0.01). By the 9th day, the surface hydrophobicity of minced meat supplemented with tapioca and ADSP was the lowest, with values of 22.36 µg and 21.22 µg, respectively, which were significantly lower than those of the other groups (*p* < 0.05).

#### 3.7.2. Carbonyl Content

In Figure 8B, a significant increase in the carbonyl content of the mince was observed with the prolongation of the storage period (*p* < 0.05). On the 9th day, the mince groups supplemented with PSP and SA showed significantly lower carbonyl content compared to the other groups, measuring 8.56 nmol/mg and 7.68 nmol/mg, respectively (*p* < 0.05). This finding contrasts slightly with the trends observed in surface hydrophobicity throughout the storage period.

#### 3.7.3. Protein Solubility

In Figure 8C, it is evident that the protein solubility in the starch-supplemented groups was higher than that in the control group; however, the difference was not statistically significant (*p* > 0.05). At 3 days of storage, the protein solubility was notably lower in the starch-added group compared to the group without starch (*p* < 0.05). After 9 days of refrigeration, the minced meat supplemented with tapioca and PSP exhibited the highest histone solubility, measuring 48.58% and 48.29%, respectively, which was significantly higher (*p* < 0.05) than the control group’s solubility of 37.69%.

#### 3.7.4. Sulfhydryl Content

In Figure 8D, it is evident that the total sulfhydryl content in minced meat decreased across all treatment groups as storage time increased. The addition of SA resulted in the highest sulfhydryl group content in meat mince during refrigeration, significantly surpassing that of the control group (*p* < 0.01). This finding suggests that SA exerted an inhibitory effect on protein oxidation, which is consistent with the results observed for surface hydrophobicity. By the 9th day, the minced meat supplemented with PSP and SA exhibited the highest sulfhydryl group content, measuring 27.57% and 26.26%, respectively, significantly exceeding that of the other groups.

#### 3.7.5. Dityrosine

As shown in Figure 8E, the dityrosine content in the minced meat groups supplemented with PSP and SA exhibited a gradually increasing trend. This suggests that the addition of a certain amount of starch was beneficial for inhibiting the oxidation of myofibrillar proteins (MP) in the minced meat, which is consistent with the observed results of carbonyl and sulfhydryl groups. In contrast, the dityrosine content in other treatment groups increased significantly over the refrigeration time, with no statistical significance in the CSP treatment group.

In conclusion, starch has a notable impact on delaying the oxidation of minced meat proteins, with SA demonstrating a particularly favorable effect in this regard.

### 3.8. Fourier Transform Infrared (FT-IR) Spectroscopy and Tertiary Structure

#### 3.8.1. Fourier Transform Infrared (FT-IR) Spectroscopy

**Figure 9:**
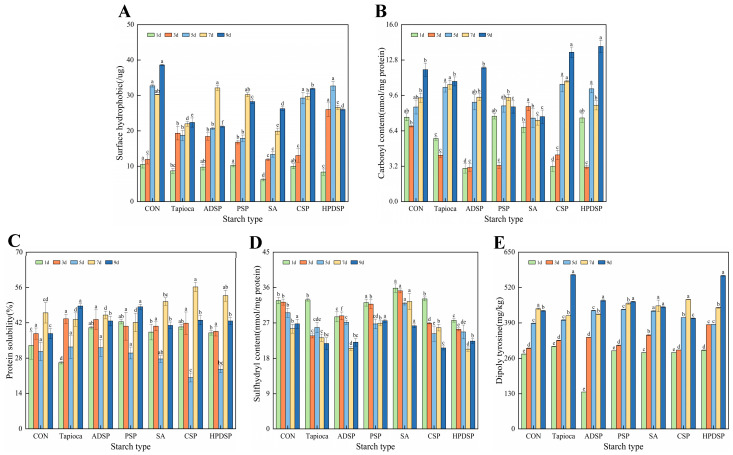
Changes in secondary structure of beef mince treated with different types of starch at 4 °C for 1 (**A**), 3 (**B**), 5 (**C**), 7 (**D**), and 9 (**E**) days, respectively. I added the explanation for lowercase letters in the figure caption.

In Figure 9, the ASDP treatment group exhibited the highest β-sheet content, peaking at 30%, along with the lowest α-helix content after 1 day of storage. The PSP treatment group reached a maximum β-sheet content of 67% after the same storage period. As storage duration increased, the β-sheet content in the PSP group decreased, while the levels of irregular curling and β-turn structures increased, indicating a transition from the β-sheet structure to irregular curling and β-turn structures over time. Additionally, the tapioca and ADSP groups also exhibited increases in β-turn and β-sheet contents.

#### 3.8.2. Tertiary Structure

**Figure 10:**
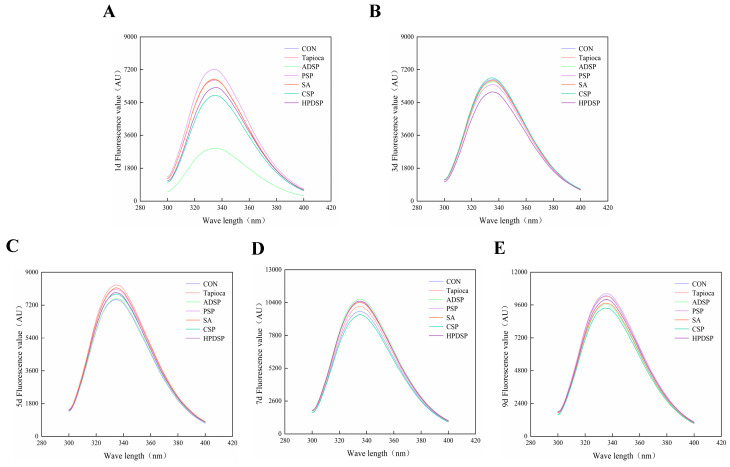
The changes in tertiary structure of beef mince treated with different types of starch at 4 °C for 1 (**A**), 3 (**B**), 5 (**C**), 7 (**D**), and 9 (**E**) days, respectively.

Regarding Figure 10, the tryptophan fluorescence intensity of minced meat in the starch-supplemented groups on the 7th and 9th days of storage was significantly higher than that observed in the same groups on the 1st, 3rd, and 5th days (*p* < 0.05). This finding suggests that the addition of starch may enhance protein folding after a certain storage period. Notably, there was no significant difference in the protein conformation of minced meat between the two groups when compared across the cold storage duration.

## 4. Discussion

### 4.1. pH and Chroma

pH is a crucial index for assessing the freshness of meat products. The pH of minced meat significantly increased with extended refrigeration time, likely due to the degradation of protein, glycogen, and other macromolecular substances into polypeptides and amino acids during enzymatic decomposition, accompanied by the release of basic groups [20], which in turn elevates the pH of the meat. Notably, the pH of minced meat with added starch was significantly higher than that of meat without starch. This is consistent with the findings presented by Mohamed Ahmed [5]. Their study demonstrated that the addition of starch did not influence the pH of the pies at the start of storage. However, an increase in the pH of all pies was observed as storage time progressed. This difference may be attributed to the accumulation of volatile compounds such as ammonia and amines produced during bacterial growth resulting from microbial enzyme activity. In meat products with lower water retention, increased surface moisture results in greater light reflection, leading to a paler muscle appearance. In the control group, both L* and a* decreased, which is consistent with Fan’s findings [21]. The decrease in L* might be a result of water release [22], while the decrease in a* can be ascribed to the oxidation of myoglobin and the subsequent formation of metmyoglobin.

### 4.2. Water-Retaining Property

The water content of ground beef gel supplemented with raw starch and modified starch was significantly higher than that of the control group. On day 9, the mince groups supplemented with tapioca and ADSP exhibited the most pronounced effect. This enhancement in water content may be attributed to the higher presence of hydrophilic groups in tapioca and ADSP, which enables them to better retain water within the ground meat [23]. Additionally, compared to the control group, the CL in the starch-added minced meat group was significantly reduced, consistent with the findings reported by Eshag Osman [5]. Their results indicated that patties made with acetylated starch exhibited the highest level of moisture retention, followed by those prepared with natural starch. This reduction may be attributed to the formation of a thin layer of starch gel on the surface of the mince, which reduces the contraction of muscle fibers and minimizes the exudation and evaporation of water during cooking [24]. Notably, the meat in the PSP group exhibited the lowest CL on day 9, likely due to its high amylopectin content. Starch-supplemented samples demonstrated a significantly higher water-binding capacity, as measured by NW levels, compared to the control group. Furthermore, the NW levels in the minced meat group supplemented with modified starch were greater than those in the group supplemented with original starch. This enhancement may be attributed to the ability of modified starch to promote the formation of a denser and more stable gel network structure within the minced meat, thereby improving its gel properties and effectively binding some of the free water present [25]. In the minced meat group supplemented with starch, WHC exhibited a marked increase compared to the control group. This is consistent with the findings of Bin Lai, which demonstrated that WHC increased proportionally with the concentration of cornstarch. This finding suggests that the addition of starch can elevate the system’s electronegativity, enhancing the electrostatic interactions between proteins. Consequently, this strengthening of protein interactions increases intermolecular binding stability and promotes the formation of a more compact and stable three-dimensional gel network [26].

Upon thawing, meat tends to lose juices, and a greater thawing loss indicates poorer stability and quality of the meat post-thawing. The reduction in freeze–thaw losses observed with the addition of starch may be attributed to the formation of a tightly ordered network [25]. Emulsification stability is a significant indicator of minced meat quality, as it reflects the product’s capacity to retain water and fat. This is typically assessed by measuring the CL of water and fat in minced meat [27], and it plays a critical role in determining the texture of the final product. Upon the addition of starch, a significant decrease in water loss from ground meat was observed, corroborating previous findings. Moreover, starch substantially reduced water release in ground meat, aligning with prior results. The incorporation of starch also led to a notable decrease in fat release from minced meat, indicating its potential to enhance lipid binding. The observed variations in total liquid release and water release with the addition of starch were comparable. Previous studies suggested that the addition of starch raises the system’s pH, which in turn boosts the electronegativity of protein molecules, enhances the interactions among protein hydrophobic groups, increases the gel network structure, and improves protein-water binding sites, thereby enhancing water retention [26].

### 4.3. Texture Profile Analysis (TPA) and Gel Strength

The hardness of the control group was notably higher than that of the meat mince group supplemented with starch. While the springiness of the meat mince group supplemented with SA was higher than that of the control group, the springiness of other starch-supplemented groups was lower than that of the control group. Furthermore, the hardness, cohesiveness, adhesiveness, and chewiness of the meat mince supplemented with starch were notably inferior compared to those of the control group. This is inconsistent with the results of Ying Shi’s study [25], in which the addition of soluble starch to Neurospora intermedia-based composite mycoprotein gel meat resulted in increased springiness, chewiness, adhesiveness, and hardness of the samples.

The mild oxidation of ground meat following the addition of starch may enhance the gel texture by promoting the formation of disulfide bonds and increasing hydrophobicity [28].

### 4.4. Dynamic Rheological Measurement

The dynamic rheological properties of minced meat with different types of starch were evaluated by measuring the G’ and G”, which reflect structural changes in the proteins [29]. The G’ exhibited a slow decrease at temperatures between 20–40 °C, primarily due to the unfolding of dissolved and swollen myofibrillar proteins resulting from the meat-mincing process under heat, which leads to the formation of a weak gel structure. In contrast, between 60 and 85 °C, the G’ increased rapidly as the proteins denatured, resulting in the reorganization of the gel network structure and a subsequent increase in the G’ [26]. The elevated G’ indicates a significant gel hardness in the minced meat, which aligns with the hardness measurements. This is mainly because the increase in temperature promotes the further aggregation and cross-linking of proteins, which change from semi-sol to elastic colloid during the heating process. The viscoelastic ground meat in the sol state was transformed into a dense and elastic gel with a network structure.

The G” of minced meat exhibits a gradual decrease between 20 and 45 °C, possibly due to the dissolution and swelling of a substantial amount of myofibrillar protein during the crushing and chopping processes, followed by protein folding during heating. This leads to a concurrent decrease in both the G’ and G”. Between 45 and 60 °C, the G’ and G” stabilize before experiencing a rapid increase from 60 to 75 °C. This temperature range is hypothesized to represent a “gel strengthening” zone, where the structural integrity and viscosity of the gel are significantly enhanced. A significant number of myosin molecules are stabilized through head disulfide bonding, hydrophobic interactions, and lateral interactions at the tail, forming a gel network characterized by high viscosity and springiness. However, this structural integrity decreases both gradually and rapidly within the temperature range of 75 to 85 °C, indicating a weakening of gel strength. The rate of protein denaturation accelerates during this phase, leading to denatured myosin tails that may increase fluidity and compromise the gel network established at lower temperatures. The G’ of ground meat is found to be higher than the G”; this is in line with the findings of Ying Shi [25], indicating that the ground meat system exhibits superior elastic characteristics upon transformation into gel, albeit retaining a weakly viscoelastic behavior [26].

### 4.5. Sensory Evaluation and Scanning Electron Microscope (SEM)

The color, taste, and springiness of beef ground gel products with added starch are superior to those of products without starch. These findings are consistent with the results of the texture analysis. Furthermore, this aligns with the findings of Mohamed Ahmed [5], who incorporated corn starch and acetylated corn starch into beef patties. The addition of natural corn starch and acetylated corn starch to the patty recipes significantly improved tenderness, juiciness, flavor, and overall acceptance compared to patties made with added animal fat (the control patties). Notable enhancements were observed in patties containing a high percentage (15%) of acetylated corn starch. This enhancement can be attributed to the ability of starch to form a stable and soft gel, which positively influences the textural properties. Consequently, the incorporation of starch results in a softer minced product that effectively mimics the mouthfeel and taste characteristics typically associated with fat in food [30]. The three-dimensional network structure of gels is a critical factor influencing their strength and water retention properties. Following starch treatment, a significant reduction in the surface pores of the mince was observed. This is consistent with the findings of Mohamed Ahmed, which indicate that the addition of 5% and 15% cornstarch can alter the microstructure of beef patties. In comparison to patties containing 5% animal fat and natural starch, those made with 5% acetylated starch exhibited a more compact gel network. This effect is likely due to the ability of starch to effectively promote protein cross-linking, resulting in a more compact network structure, which aligns with the observed gel strength results. The primary mechanisms behind this phenomenon are hydrophobic interactions and hydrogen bonding between starch and meat proteins [31]. This finding is consistent with the work of Fan et al. [32], who also noted that the micronetwork structure of grass carp myofibrin gel could be enhanced through the use of ADSP. The observed improvement is primarily attributed to the filling effect associated with starch gelatinization and swelling. The introduction of cross-linked bonds by tapioca at the outset facilitates the formation of a weak network, which subsequently evolves into an interpenetrating network during the heating process. This phenomenon results in a denser microstructure of the minced meat [33]. Furthermore, the swelling capacity of ADSP is enhanced following acetylation modification [34], which may lead to the compression of the protein network structure and potential coarsening due to excessive expansion during heating. This observation is in agreement with the findings of Sun et al. [33], who demonstrated that the combination of grass carp myofibrillar protein with cross-linked modified starch yields a finer microstructure.

### 4.6. Lipid Oxidation Index

In the evaluation of primary oxidizing compounds in foods, the POV is frequently utilized [35]. As the storage time increased, the POV of minced meat also increased. The observed alterations in POV observed indicate the presence and formation of H_2_O_2_ lipid hydroperoxides. However, due to the inherent instability of primary oxidation products during their formation, relying solely on the POV for evaluating the extent of meat oxidation may be inadequate [36]. Hence, measuring the TBARS in minced meat provides further insights into lipid oxidation, as the TBARS serves as a significant indicator of malondialdehyde (MDA) content. MDA is a crucial byproduct of lipids and a fundamental marker of this process [35]. Over the prolonged storage duration, TBARS levels in minced meat exhibited a rising trend. This increase in TBARS levels could be attributed to lipid oxidation and the subsequent decomposition of malondialdehyde into organic acids and alcohols, as well as interactions with other constituents of meat, such as glucose, proteins, and amino acids [37].

### 4.7. Protein Oxidation Index

In the starch-adding group, the surface hydrophobicity of the meat was lower than that in the control group. This reduction may be attributed to the addition of starch, which can inhibit the exposure of hydrophobic groups, thereby reducing hydrophobic aggregation among protein molecules [38]. During the cold storage of minced meat, the exchange of sulfhydryl–disulfide groups plays a crucial role in protein crosslinking and aggregation [39]. With extended storage time, the total sulfhydryl content in all treatment groups of minced meat decreased. This decline may be linked to the formation of disulfide-dependent soluble aggregates in meat mince due to refrigeration and starch treatment [38]. In the starch group, the surface hydrophobicity of the meat was lower than that of the control group. This reduction may be attributed to the addition of starch, which can inhibit the exposure of hydrophobic groups and consequently limit hydrophobic aggregation among protein molecules.

As the storage period increases, the overall carbonyl content of the mince exhibited a significant upward trend (*p* < 0.05). The increase in carbonyl content can be attributed to oxidation-induced peptide cleavage and the conversion of specific amino acid residues into carbonyl derivatives [40]. Protein solubility is a crucial indicator for assessing protein functionality, as a decrease in solubility may coincide with a decline in protein activity [41]. In each starch-added group, the protein solubility was higher than that in the control group, which may be attributed to the protective effect of starch that shields proteins from oxidative damage and influences their structural folding [42]. During the cold storage of minced meat, the exchange of sulfhydryl–disulfide plays a crucial role in protein crosslinking and aggregation [39]. With the extension of storage time, the total sulfhydryl content in all treatment groups of minced meat decreased, likely due to the formation of disulfide-dependent soluble aggregates during refrigeration and starch treatment [38]. When a protein’s side chain is targeted by reactive oxygen species (ROS), it generates tyrosine free radicals. These radicals, which can occur on either the same or different protein chains, readily combine to form dityrosine, leading to the intramolecular or intermolecular crosslinking of proteins. Dityrosine, a fluorescent amino acid, exhibits a fluorescence intensity that is directly linked to the level of protein oxidation. The observed reduction in dityrosine fluorescence intensity in minced meat treated with PSP and SA may be attributed to the enhancement of muscle’s capacity to scavenge free radicals, facilitated by these two starches [43].

### 4.8. Fourier Transform Infrared (FT-IR) Spectroscopy and Tertiary Structure

Changes in the amide I region are commonly utilized to indicate alterations in the secondary structure of proteins. This region is primarily associated with the in-plane bending of the N-H group in the peptide chain, the stretching vibration of the C=O group, and the stretching vibration of C-N in the peptide bond. The amide I band consists of overlapping bands near 1650–1660 cm^−1^, 1660–1665 cm^−1^, 1665–1680 cm^−1^, and above 1680 cm^−1^. They represent the α-helix structure, random coil structure, β-sheet structure, and β-sheet structure, respectively [29]. Consistent with Sun’s findings [33], the increased presence of β-turn and β-sheet in the minced meat group supplemented with tapioca and ADSP aligns with their observations regarding the effects of tapioca and denatured starch in enhancing the expansion of α-helix and the formation of β-sheet. Additionally, Zhang et al. [44] highlighted that the reduction in α-helix content signifies structural expansion of proteins, while the increase in β-sheet indicates protein aggregation, thereby facilitating the development of a refined network protein structure.

The absorption of ultraviolet light and fluorescence emission are properties exhibited by the side chain groups of aromatic amino acid residues. Specifically, tryptophan residues are excited at a wavelength of 280 nm, which enables the characterization of spatial conformational changes in proteins through fluctuations in fluorescence intensity [26]. A higher tryptophan fluorescence intensity indicates that the protein is in a folded state, while a lower fluorescence intensity indicates that the protein is in a partially or fully unfolded state. The fluorescence intensity of tryptophan in meat proteins from minced meat with added starch at 7 and 9 days of storage was significantly higher than that observed in minced meat with added starch at 1, 3, and 5 days of storage. This increase may result from the MPs adopting a more relaxed conformation due to starch treatment during cold storage, thereby exposing previously buried amino acid residues [45].

## 5. Conclusions

Starch has been shown to enhance the edible quality of beef mince gel products during cold storage. The inclusion of starch significantly improves both the color and water retention of the mince, with particular emphasis on the positive effects of PSP and HPDSP, which exhibit the most notable enhancement in water retention. Furthermore, the addition of starch positively influences the texture of the meat, increases the gel strength, enhances sensory attributes, and optimizes the microstructure of the mince. To a certain degree, the incorporation of starch also serves to inhibit lipid and protein oxidation in beef mince.

The application of various types of starch in meat products holds significant research importance, and its potential future applications are promising. This includes the direct incorporation of starch into meat products, the development of starch-based packaging films, and the use of starch as a fat substitute in meat formulations. These approaches represent effective strategies for improving the quality of meat products during storage.

## Figures and Tables

**Table 1:** Sensory evaluation table.

Item	Standard	Mark
Color and luster	White, dull, yellow	1 10
Taste	Coarse taste, strong powdery feeling	1 10
Masticatory	Chew 1–5 times, and the product will become meat residue Chew more than 10 times, the product will become meat residue	1 10
Organizational structure	The surface of the section is uneven, there are pores, and the tissue is loose The surface of the section is flat, without porosity, and the tissue is compact	1 10
Elasticity	It does not recover after finger pressure Recover immediately after pressing with your fingers	1 10

**Table 2:** Effect of cold storage time on hardness, springiness, cohesiveness, adhesiveness, and chewiness of meat minced with different kinds of starch.

Starch Type	Number of Days	Hardness/g	Springiness	Cohesiveness	Adhesiveness/g.s	Chewiness
CON	1	2493 ± 91 ^a^	0.83 ± 0.01 ^a^	0.69 ± 0.01 ^a^	1716 ± 61 ^a^	1424 ± 51 ^a^
	3	2789 ± 26 ^a^	0.81 ± 0.01 ^a^	0.70 ± 0.01 ^a^	1957 ± 26 ^a^	1591 ± 18 ^a^
	5	2871 ± 49 ^a^	0.81 ± 0.01 ^a^	0.71 ± 0.01 ^a^	2042 ± 18 ^a^	1658 ± 44 ^a^
	7	2899 ± 15 ^a^	0.77 ± 0.01 ^b^	0.72 ± 0.01 ^a^	2251 ± 114 ^a^	1746 ± 20 ^a^
	9	2954 ± 45 ^a^	0.73 ± 0.01 ^b^	0.73 ± 0.01 ^a^	2357 ± 81 ^a^	1816 ± 20 ^a^
Tapioca	1	2191 ± 43 ^a^	0.82 ± 0.03 ^a^	0.58 ± 0.05 ^b^	1274 ± 128 ^b^	1048 ± 138 ^b^
	3	2218 ± 90 ^a^	0.72 ± 0.01 ^b^	0.58 ± 0.01 ^b^	1290 ± 75 ^b^	931 ± 61 ^b^
	5	1908 ± 54 ^c^	0.77 ± 0.07 ^b^	0.51 ± 0.06 ^b^	963 ± 102 ^c^	746 ± 154 ^b^
	7	2555 ± 125 ^a^	0.82 ± 0.01 ^a^	0.69 ± 0.04 ^a^	1749 ± 12 ^b^	1440 ± 22 ^a^
	9	1998 ± 197 ^b^	0.76 ± 0.05 ^b^	0.52 ± 0.03 ^c^	1040 ± 81 ^b^	792 ± 32 ^c^
ADSP	1	872 ± 39 ^d^	0.65 ± 0.02 ^c^	0.46 ± 0.05 ^c^	401 ± 9 ^c^	260 ± 12 ^d^
	3	1072 ± 49 ^b^	0.74 ± 0.05 ^b^	0.49 ± 0.01 ^c^	525 ± 48 ^c^	390 ± 60 ^c^
	5	1576 ± 19 ^c^	0.74 ± 0.04 ^b^	0.48 ± 0.06 ^c^	763 ± 61 ^c^	565 ± 64 ^c^
	7	1534 ± 116 ^c^	0.72 ± 0.14 ^b^	0.62 ± 0.04 ^b^	940 ± 59 ^c^	672 ± 111 ^c^
	9	1568 ± 49 ^c^	0.79 ± 0.03 ^b^	0.56 ± 0.03 ^c^	883 ± 29 ^c^	696 ± 21 ^c^
PSP	1	1825 ± 39 ^b^	0.80 ± 0.02 ^a^	0.58 ± 0.03 ^b^	1057 ± 53 ^b^	842 ± 61 ^b^
	3	2538 ± 78 ^a^	0.77 ± 0.04 ^b^	0.58 ± 0.03 ^b^	1474 ± 28 ^b^	1138 ± 72 ^b^
	5	1877 ± 54 ^c^	0.74 ± 0.02 ^b^	0.52 ± 0.01 ^b^	984 ± 39 ^c^	726 ± 16 ^b^
	7	2618 ± 70 ^a^	0.77 ± 0.04 ^b^	0.68 ± 0.02 ^b^	1786 ± 40 ^b^	1378 ± 61 ^b^
	9	2672 ± 94 ^a^	0.80 ± 0.03 ^a^	0.67 ± 0.02 ^b^	1780 ± 98 ^c^	1433 ± 131 ^b^
SA	1	1880 ± 36 ^b^	0.86 ± 0.02 ^a^	0.65 ± 0.05 ^a^	1231 ± 96 ^b^	1054 ± 107 ^b^
	3	2229 ± 63 ^a^	0.85 ± 0.04 ^a^	0.61 ± 0.05 ^a^	1369 ± 145 ^b^	1049 ± 28 ^a^
	5	2362 ± 33 ^b^	0.83 ± 0.03 ^a^	0.58 ± 0.06 ^b^	1361 ± 134 ^b^	1138 ± 151 ^b^
	7	2210 ± 25 ^a^	0.84 ± 0.04 ^a^	0.68 ± 0.02 ^b^	1500 ± 66 ^b^	1260 ± 110 ^b^
	9	2553 ± 44 ^a^	0.80 ± 0.02 ^a^	0.56 ± 0.03 ^c^	1440 ± 93 ^b^	1159 ± 106 ^b^
CSP	1	1570 ± 61 ^c^	0.77 ± 0.02 ^b^	0.60 ± 0.01 ^a^	949 ± 32 ^c^	731 ± 46 ^c^
	3	1889 ± 70 ^b^	0.79 ± 0.03 ^a^	0.60 ± 0.02 ^a^	1131 ± 27 ^b^	890 ± 14 ^b^
	5	2059 ± 25 ^b^	0.80 ± 0.05 ^ab^	0.61 ± 0.02 ^a^	1258 ± 31 ^b^	1008 ± 35 ^b^
	7	2553 ± 84 ^a^	0.81 ± 0.01 ^a^	0.67 ± 0.03 ^b^	1714 ± 119 ^b^	1391 ± 116 ^b^
	9	2167 ± 100 ^b^	0.82 ± 0.04 ^a^	0.71 ± 0.03 ^a^	1531 ± 92 ^b^	1257 ± 130 ^b^
HPDSP	1	1242 ± 76 ^c^	0.73 ± 0.05 ^b^	0.53 ± 0.03 ^b^	660 ± 66 ^c^	486 ± 72 ^d^
	3	1579 ± 78 ^c^	0.80 ± 0.06 ^a^	0.64 ± 0.06 ^a^	1017 ± 87 ^b^	818 ± 121 ^b^
	5	2335 ± 18 ^b^	0.83 ± 0.03 ^a^	0.68 ± 0.01 ^a^	1579 ± 19 ^b^	1305 ± 59 ^c^
	7	1850 ± 14 ^b^	0.73 ± 0.03 ^b^	0.55 ± 0.05 ^c^	1021 ± 90 ^c^	746 ± 99 ^c^
	9	1710 ± 94 ^c^	0.76 ± 0.06 ^b^	0.56 ± 0.04 ^c^	951 ± 97 ^c^	726 ± 121 ^c^

Note: Values are the average of values in triplicate ± SD. Different letters indicated significant differences between samples treated with different starch on the same day (*p* < 0.05).

## Data Availability

The original contributions presented in the study are included in the article, further inquiries can be directed to the corresponding author.
